# P-467. Prevalence of Meningococcal Carriage by Serogroup in Adolescents: a Global Systematic Literature Review

**DOI:** 10.1093/ofid/ofaf695.682

**Published:** 2026-01-11

**Authors:** Zeki Kocaata, Lucian Gaianu, Ifeanyi Ubamadu, Laura Taddei, Thatiana Pinto, Hiral Shah, Pavo Marijic, Patrice Carter, Matthew Turner, Andrew Easton

**Affiliations:** GSK, Wavre, Brabant Wallon, Belgium; GSK, Wavre, Brabant Wallon, Belgium; GSK, Wavre, Brabant Wallon, Belgium; GSK, Wavre, Brabant Wallon, Belgium; GSK, Wavre, Brabant Wallon, Belgium; GSK, Wavre, Brabant Wallon, Belgium; GSK, Wavre, Brabant Wallon, Belgium; Health Economics & Outcomes Research Ltd, Cardiff, Wales, United Kingdom; Health Economics & Outcomes Research Ltd, Cardiff, Wales, United Kingdom; Health Economics & Outcomes Research Ltd, Cardiff, Wales, United Kingdom

## Abstract

**Background:**

Adolescents and young adults are the primary reservoir for *Neisseria meningitidis*, with high carriage rates and social behaviours that aid transmission. This age group represents an important proportion of outbreak cases, and plays a prominent role in transmission in the community. However, evidence is lacking on which meningococcal serogroups are carried, and risk factors for carriage. The aim of this global systematic literature review (SLR) was to assess meningococcal carriage prevalence by serogroup, age, and region, and key factors associated with carriage.

**Figure:**
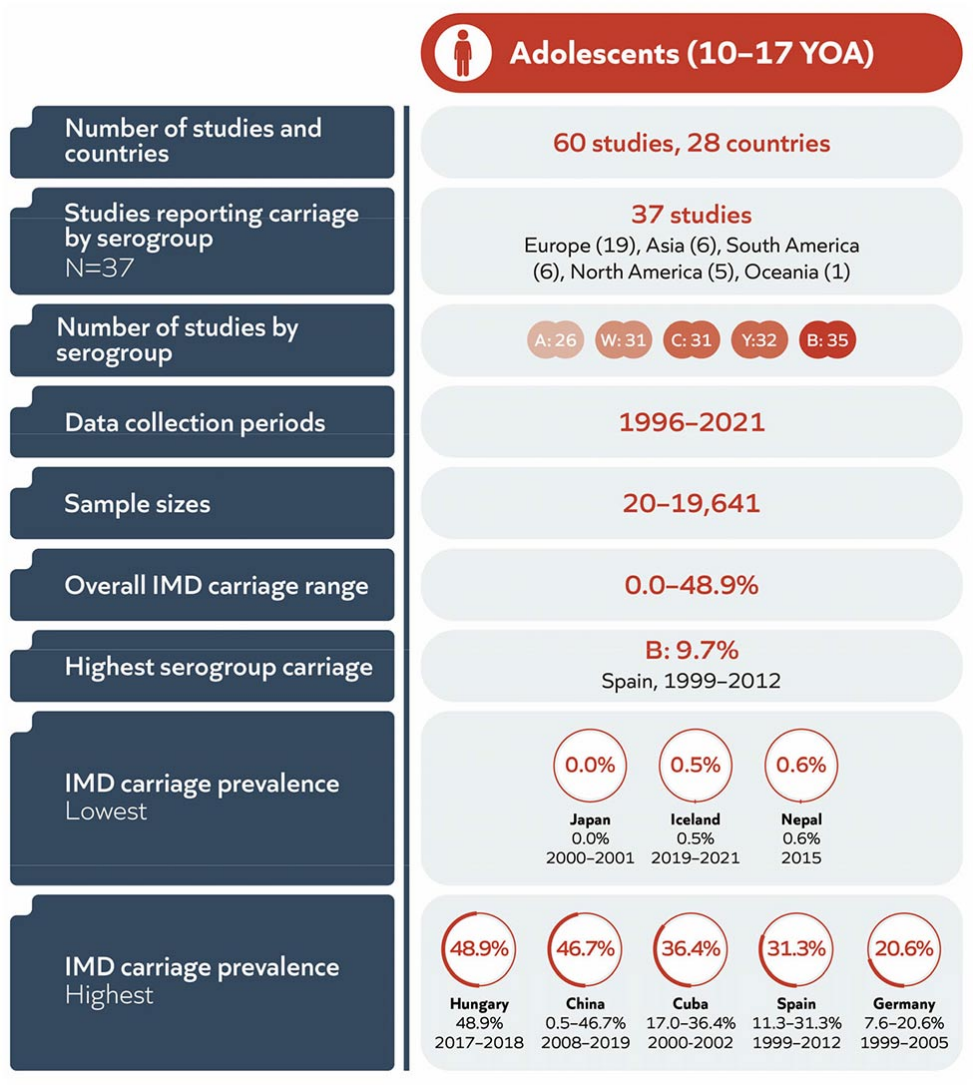
Summary of Results YOA : Years of age

**Methods:**

An SLR was conducted using Medline, Embase, and public health websites to identify studies on age- and serogroup-specific meningococcal carriage prevalence (January 2000 to April 2024, no language restrictions) following PRISMA and Cochrane guidelines. Studies from the African meningitis belt and on household/case contacts were excluded. Findings are reported for adolescents 10–17 years of age (YOA).

**Results:**

Among the 203 studies included in the SLR (all ages), 60 studies reported carriage prevalence in adolescents (37 with serogroup data), with wide sample sizes (20–19,641) and timeframe ranges (1996–2021). Serogroup B carriage was reported most frequently (in 35/37 serogroup-specific studies). Overall carriage prevalence among adolescents varied across countries, ranging from 0.0% (Japan, 2000–2001) to 48.9% (Hungary, 2017–2018), with the highest serogroup-specific prevalence reported for serogroup B (9.7%) (Figure 1).

Key factors associated with carriage in adolescents included smoking, attending social gatherings, kissing, male gender, white ethnicity, and lower parental education.

**Conclusion:**

In adolescents, serogroup B carriage was the most commonly reported, and with the highest serogroup-specific carriage rates, highlighting the potential disease risk caused by this serogroup. Carriage was associated with lifestyle and various other factors. Understanding the magnitude and risk factors for carriage can help to better understand transmission and disease risk, and develop public health interventions to prevent subsequent disease in this population.

**Disclosures:**

Zeki Kocaata, PhD, GSK: Employee|GSK: Stocks/Bonds (Public Company) Lucian Gaianu, MSc, GSK: Employee Ifeanyi Ubamadu, MSc, GSK: Employee|GSK: Stocks/Bonds (Private Company) Laura Taddei, M.Sc, GSK: employee|GSK: Stocks/Bonds (Private Company) Thatiana Pinto, PhD, GSK: employee|GSK: Stocks/Bonds (Public Company) Hiral Shah, PhD, GSK: Employee|GSK: Stocks/Bonds (Public Company) Pavo Marijic, PhD, GSK: employee|GSK: Stocks/Bonds (Public Company) Patrice Carter, PhD, GSK: Grant/Research Support Matthew Turner, PhD, GSK: Grant/Research Support Andrew Easton, MSc, GSK: Grant/Research Support

